# How Cowpox Virus Turns Back Cell Defenses

**DOI:** 10.1371/journal.pbio.1001431

**Published:** 2012-11-27

**Authors:** Caitlin Sedwick

**Affiliations:** Freelance Science Writer, San Diego, California, United States of America

**Figure:**
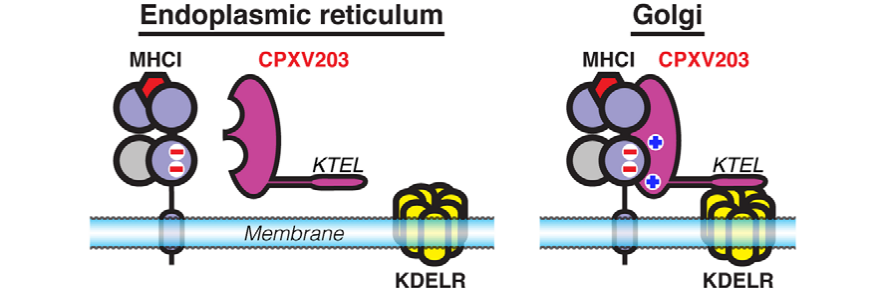
Cowpox-encoded CPXV203 subverts antigen presentation by engaging MHCI in the Golgi and subjecting it to KDEL receptor mediated retrieval.

Viruses follow a well-choreographed battle plan to mount an infection. First, they invade cells, then stage a takeover of its genetic and protein replication machinery. Once the virus assumes control, it forces the cell to create thousands of copies of the virus, which usually ends up killing the cell. Organisms have evolved elaborate defenses to prevent viruses from entering cells in the first place or, as a last resort, to kill their own infected cells in order to prevent the infection from spreading. But viruses have also evolved countermeasures to these defense mechanisms. In this issue of *PLOS Biology*, William McCoy, David Fremont, and colleagues explain how a countermeasure devised by cowpox virus works to help the virus establish and maintain its presence in an infected cell.

Cowpox virus can infect and replicate in the cells of many different mammalian species, including humans. One of the keys to cowpox's virulence is a viral protein called CPXV203, which acts by interfering with a major component of the body's antiviral defense apparatus, the major histocompatibility class I (MHCI) proteins.

MHCI proteins serve as a surveillance device. When a virus invades a cell and begins producing viral proteins, some of these proteins are fed into the proteasome where they are chopped into short peptide fragments. These viral peptides are then transported from the cytosol into the endoplasmic reticulum (ER), where immature MHCI proteins await in the embrace of quality control chaperones. Peptide loading onto MHCI allows mature assembly, at which point the complex is shuttled to the Golgi, where it is subjected to further quality-control testing. MHCI proteins with loosely bound peptides are recycled back to the ER to find one with a better fit. Meanwhile, those with tightly bound peptides are sent on to the cell surface, where they display their catch to cells of the immune system. When the immune cells detect MHCI molecules displaying viral peptides, they kill the infected cell to stop the release of more viruses. But CPXV203 somehow prevents MHCI molecules from ever reaching the cell surface, leaving the immune system ignorant of the infection. How it manages this wasn't clear, so McCoy and colleagues set out to investigate.

Other groups had studied CPXV203 and concluded that it did not interfere with cells' ability to get viral peptides into the ER or to bind peptides onto MHCI proteins. Consistent with this latter finding, the authors found that CPXV203 binds solely to MHCI molecules that are in their mature form—that is, after peptides have been matched with MHCI molecules.

Having found that CPXV203 binds to mature MHCI molecules, the authors tried to characterize this interaction further. They first measured the binding affinity between CPXV203 and several different kinds of MHCI molecules (including some from different species), and found that CPXV203 was able to bind to each of these MHCI proteins. This broad affinity for MHCI molecules of different origins reflects, and may even partly explain, cowpox virus' ability to thrive in the cells of different species.

But McCoy and colleagues also noticed a curious thing: in every case, the interaction between CPXV203 and MHCI proteins was fairly weak. Because pH can frequently affect the affinity of protein-protein interactions, the authors next tested CPXV203-MHCI binding under different pH conditions. Strikingly, binding was enhanced 50-fold when pH was adjusted to mimic the conditions that prevail in the Golgi, suggesting that tight CPXV203-MHCI interaction occurs primarily in the Golgi.

To explore how CPXV203 binds to MHCI proteins, the authors examined the structure of the CPXV203-MHCI complex. The structure identified the CPXV203 residues responsible for enhanced complex stability in the low pH of the Golgi. It also demonstrated that CPXV203 attaches to portions of the MHCI molecules that are highly conserved, and which only appear only after MHCI achieves its mature form. This interaction with conserved MHCI regions explains why CPXV203 can bind to MHCI proteins from different species, and also why it is so effective at its job; hosts can't acquire mutations at these sites without risking the proper function of the protein. Importantly, mutation of amino acid residues (in either CPXV203 or MHCI) predicted by the crystal structure to be critical for their interaction, indeed prevents their association and allows MHCI proteins to reach the cell surface.

Altogether, the authors' data suggest that CPXV203 binding to mature MHCI in the Golgi apparatus prevents MHCI from reaching the cell surface. Consistent with this, other groups had previously shown that CPXV203 possesses a motif that engages a chaperone retrieval system, the KDEL receptor, which traffics proteins from the Golgi to the ER. McCoy et al. therefore argue that CPXV203 works by subverting this quality control pathway in the Golgi.

Ultimately, this work provides important insights into how CPXV203 operates. It also shows how CPXV203 cooperates with other virally encoded countermeasures to help cowpox virus propagate within its hosts.

**McCoy WH, Wang X, Yokoyama WM, Hansen TH, Fremont DH (2012) Structural Mechanism of ER Retrieval of MHC Class I by Cowpox. doi:10.1371/journal.pbio.1001432**

